# A Systematic Review on Sociodemographic, Financial and Psychological Factors Associated with COVID-19 Vaccine Booster Hesitancy among Adult Population

**DOI:** 10.3390/vaccines11030623

**Published:** 2023-03-09

**Authors:** Shruti Ayyalasomayajula, Aditi Dhawan, Mohammed Salim Karattuthodi, Shabeer Ali Thorakkattil, Suhaj Abdulsalim, Mohamed Hassan Elnaem, Sathvik Sridhar, Mazhuvancherry Kesavan Unnikrishnan

**Affiliations:** 1 Department of Pharmacy Practice, Manipal College of Pharmaceutical Sciences, Manipal Academy of Higher Education, Madhav Nagar 576104, India; 2 Pharmacy Services Department, Johns Hopkins Aramco Healthcare, Dhahran 34465, Saudi Arabia; 3 Department of Pharmacy Practice, Unaizah College of Pharmacy, Qassim University, Buraydah 52571, Saudi Arabia; 4 School of Pharmaceutical Sciences, Universiti Sains Malaysia, George Town 11800, Malaysia; 5 Department of Clinical Pharmacy & Pharmacology, RAK College of Pharmacy, RAK Medical and Health Sciences University, Ras Al Khaimah 11172, United Arab Emirates; 6 Department of Pharmacy Practice, Nitte Gulabi Shetty Memorial Institute of Pharmaceutical Sciences, Mangalore 575018, India

**Keywords:** COVID-19 booster dose, vaccine booster hesitance(VBH), hesitancy factors, reluctance among adults

## Abstract

Background: While considerable evidence supports the safety and efficacy of COVID-19 vaccines, a sizable population expresses vaccine hesitancy. As per the World Health Organization, vaccine hesitancy is one of the top 10 hazards to global health. Vaccine hesitancy varies across countries, with India reporting the least vaccine hesitancy. Vaccine hesitancy was higher toward COVID-19 booster doses than previous shots. Therefore, identifying factors determining COVID-19 vaccine booster hesitance (VBH) is the *sine qua non* of a successful vaccination campaign. Methodology: This systematic review followed Preferred Reporting Items for Systematic Reviews and Meta-analysis (PRISMA) 2020 standards. A total of 982 articles were pooled from Scopus, PubMed and Embase, while 42 articles that addressed the factors of COVID-19 VBH were finally included for further analysis. Result: We identified factors responsible for VBH and divided them into three major groups: sociodemographic, financial, and psychological. Hence, 17 articles stated age to be a major factor for vaccine hesitancy, with most reports suggesting a negative correlation between age and fear of poor vaccination outcomes. Nine studies found females expressing greater vaccine hesitancy than males. Trust deficit in science (n = 14), concerns about safety and efficacy (n = 12), lower levels of fear regarding infection (n = 11), and worry about side effects (n = 8) were also reasons for vaccine hesitancy. Blacks, Democrats, and pregnant women showed high vaccine hesitancy. Few studies have stated income, obesity, social media, and the population living with vulnerable members as factors influencing vaccine hesitancy. A study in India showed that 44.1% of vaccine hesitancy towards booster doses could be attributed dominantly to low income, rural origin, previously unvaccinated status, or living with vulnerable individuals. However, two other Indian studies reported a lack of availability of vaccination slots, a lack of trust in the government, and concerns regarding safety as factors for vaccine hesitancy toward booster doses. Conclusion: Many studies have confirmed the multifactorial nature of VBH, which necessitates multifaceted, individually tailored interventions that address all potentially modifiable factors. This systematic review chiefly recommends strategizing the campaign for booster doses by identifying and evaluating the reasons for vaccine hesitancy, followed by appropriate communication (at both individual and community levels) about the benefits of booster doses and the risk of losing immunity without them.

## 1. Introduction

Vaccines have a remarkable track record of reducing life-threatening infections. Large scale COVID-19 vaccination programs flattened the unprecedented pandemic [1] but fell short of its true potential because of suboptimal availability and muted acceptance. The high prevalence of vaccine refusal or hesitancy significantly dampened herd immunity [2]. Despite considerable evidence supporting the efficacy and safety of COVID-19 vaccines, the majority are still reluctant to vaccinate [3].

The World Health Organization (WHO) regards vaccine hesitancy as one of the top ten hazards to world health, and they define vaccine hesitancy as “a delay in acceptance or refusal of vaccination despite the availability of vaccination services” [4]. More than 10 million deaths could have been prevented by vaccination between 2010 and 2015, amply demonstrating the prevailing vaccine hesitancy. While the COVID-19 vaccine was developed and distributed at an unprecedented pace, mounting skepticism and vaccine hesitancy continue to pose a challenge. COVID-19 vaccine hesitancy varies by country: Kuwait (76%), Jordan (71%), Russia (45%), Poland (44%), France (41%), the United Kingdom (25%), and the United States (21%) [5]. Hesitancy and refusal are different; vaccine-hesitant people do not refuse vaccination completely but delay doses either entirely or partially [6]. Therefore, the hesitation rate may not be complementary to the acceptance rate. Compared to developed nations, the acceptance rate for COVID-19 vaccines were higher in lower and middle-income (LMIC) countries. The average acceptance rate in India was 84%, followed by Pakistan and Burkina Faso (66.5%), the United States (64.6%), and Russia (30.4%) [7].

Acceptance levels may be lower for newer vaccines than for more familiar older vaccines. COVID-19 booster doses, despite their availability and being a pre-requisite for continued COVID-19 protection, are not widely accepted in society [8]. The unpredictable trajectory of the COVID-19 pandemic and the uneven introduction and coverage of COVID-19 vaccines elicited multiple studies identifying reasons for vaccine hesitancy toward booster shots in different parts of the world. Although India accounts for very few studies highlighting the same, we hypothesize that the factors resulting from these Indian scenarios would be uniquely different due to a vast population. Therefore, it was necessary to recognize and analyze the reasons for VBH in India’s highly diverse, multiethnic, multilingual, multicultural society.

To break the chain and ensure health, we need to detect and correct issues leading to COVID-19 VBH. This systematic review proposes to identify, compare, and evaluate factors that influence VBH in India and the world.

## 2. Materials and Methods

This study focuses on pinpointing the various factors for VBH among adults in India and globally. The systematic review was conducted following Preferred Reporting Items for Systematic Reviews and Meta-analysis (PRISMA) 2020 standards [9].

### 2.1. Search Strategies and Information

Articles specific to the study objectives were retrieved from PubMed, Scopus, and Embase, using the medical subject headings (MeSH) terms, “booster dose OR vaccine booster OR booster shot OR third dose OR additional dose OR vaccine continuation OR precautionary dose”, “SARS-CoV-2 OR COVID-19 OR corona OR corona virus OR coronaviruses OR coronavirus”, “hesitance OR hesitancy OR acceptance OR acceptancy OR reluctance OR unwillingness”, incorporating “AND” Boolean operators. Moreover, those articles with randomized clinical trials, cohort, cross-sectional study designs were included. We did not consider, unpublished data, abstracts from conferences, proposed models, case reports and series, and review papers.

### 2.2. Study Workflow and Criteria

We included systematic reviews, meta-analyses, randomized clinical trials, and cohort studies that focused on various factors behind VBH, described both globally and in India, and published in English between January 2021 and October 2022.

### 2.3. Selection of Study, Data Retrieval and Quality Checks

Two independent researchers separately identified and screened the titles and abstracts of the retrieved articles. Next, individual papers were subjected to full-text review. Conflicts in opinion between the two authors were resolved by the third and fourth.

The prime reasons for VBH are presented in Supplementary Data S1. We used the Critical Appraisal Skills Program (CASP) checklist to evaluate each selected article [10].

## 3. Results

### 3.1. Description of Search Criteria

From a total of 982 articles, 690 relevant articles were shortlisted upon removal of duplicates. Considering the exclusion criteria, 601 articles were removed, and the remaining 89 were eligible for full-text evaluation. Further, 42 articles that addressed factors relevant to the COVID-19 VBH were included in the review.

### 3.2. Factors Affecting COVID-19 Booster Vaccine Hesitancy

**Figure 1:**
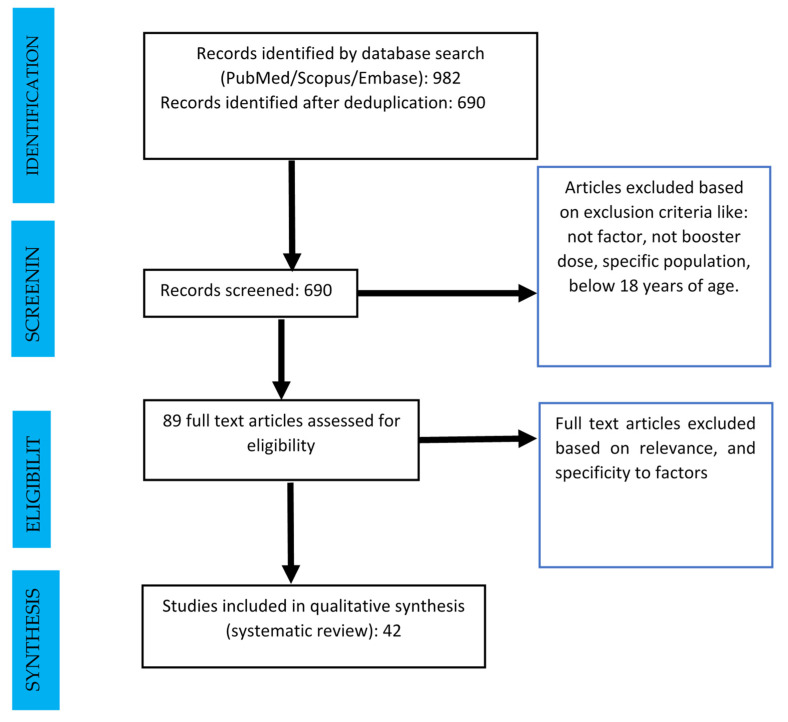
The workflow of the study utilizing the PRISMA guideline.

Factors influencing the VBH were subdivided into those from and outside India (Figure 1).

#### 3.2.1. India

We retrieved three studies from India that identified factors contributing to vaccine hesitancy.

Geetanjali C. Achrekar et al. [11], in a cross-sectional study using an online survey over two months, reported that 44.1% of respondents expressed VBH, including those who did not avail of the primary dose of the vaccine, had an annual income of less than 2.96 lakhs INR, were rural residents living without family/friends infected with COVID-19, associated a booster dose with side effects, and lived with non-vulnerable members. However, in Masthi NR R et al.’s [12] six-month study, participants expressed VBH because they were not due for it, followed by unavailability of the vaccine or vaccination slot on the CoWIN portal (Indian government vaccination registration website). Other reasons listed were: “COVID-19 did not exist any longer”, “didn’t trust the government”, and “the booster dose wasn’t necessary”. Nevertheless, Sajith Vellappally et al. [13] reported that the most relevant factors behind VBH in India were concerns with vaccine efficacy and fear of long-term side effects.

#### 3.2.2. Other Than India

**Figure 2:**
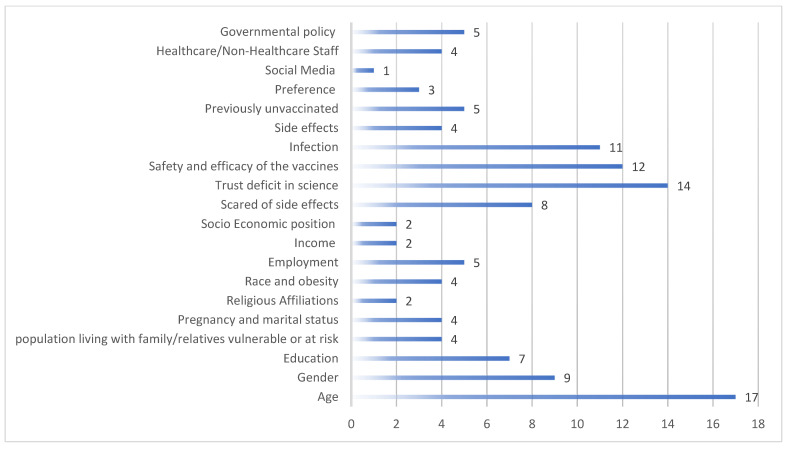
Graphical representation of number of articles attributing factors responsible for VBH.

Forty studies in different countries outside India listed multiple factors for VBH. These were specific to sociodemographics such as age, gender, education, marital status and members associated, religious affiliation, race, lifestyle and economical aspects, psychological, infection, type of vaccine, social media, professional background, and government policy-related particulars (Figure 2).

##### Sociodemographic Factors

Qin et al. [14] observed that the VBH was higher among people aged ≥ 50 years (81.7% acceptance) than those aged 21–30 years (94.6% acceptance), which is supported by Nguyen KH et al. [15] and Stephen R et al. Accordingly, younger age inversely correlated with VBH. However, Petros Galanis et al. [16] reported that fear of poor vaccination outcomes was negatively correlated with age as supported by Yadete et al. [17], Abouzid et al. [18], Batra et al. [19], Neil G bennett et al. [20], Rzymski et al., Wirawan et al. [21], Elise Paul et al. [22], and Xiaoxiao wang et al. [23]. Additionally, Lounis et al. [24] found that younger age (<35 year) groups were 1.42 times more likely to express VBH than the older age (≥65 years) groups, as also supported by Mostafa et al. [25].

Nevertheless, respondents between the age of 25 and 54 years expressed the least VBH as per Jakob Weitzer et al. [26]. According to Klugar et al. [27], the median age for all acceptor groups was 29 years. Kowalski E et al. observed substantial variation in VBH between young and middle aged and young and old, but not between middle-aged and old. According to Ruitong Wang et al. [28], 25–44 year old expressed 3.42 times more VBH than those between 18–24 years of age. Abdul Moeed et al.’s [29] study showed that children under 18 years expressed maximum VBH, including teenagers unsure about vaccine effectiveness. Kowalski E et al. [30] observed substantial variation in VBH between young and middle aged and young and old, but not between middle-aged and old.

Reports by Fan Wu et al. [31] and Mohamed Abouzid et al. [18] females expressed substantially more VBH than males (19.5% men vs. 27.6% women; *p* < 0.001). Similar observations were reported by Thin Mon Kyaw et al. [32], Elias Kowalski et al. [30], Tesfaye Yadete et al. [17] (55.4% females hesitancy), Mohamed Abouzid et al. [18] (19.5% men vs. 27.6% women refused to take vaccine), Xiaoxiao Wang et al. [23] (80.2% male acceptance vs. 72.2% female acceptance), and Miloslav Klugar et al. [27] (male 79.3% acceptance vs. female 69.7% acceptance).

While Piotr Ryzmyski et al. [33] found females less prone to VBH, Sameh Attia et al. [34] found no significant difference in VBH between genders. However, LGBTQ + participants (25%) were more inclined to express VBH than females (7.2%) and males (8.2%).Less educated populations expressed greater VBH, according to Tesfaye Yadete et al. [17], Kavita Batra et al. [11], Kimberly H. Nguyen et al. [15], and Elise Paul et al. [22]. However, Fan Wu et al. [31] portrayed lowest VBH among those with junior school education. Surprisingly, those with tertiary education expressed greater VBH, according to Kevin Y. K. Tan et al. [35].

Participants living with a vulnerable family member or have a family member or relative with a history of COVID-19 with comorbidities expressed less VBH, as reported by Tesfaye Yadete et al. [17] and Ammar Abdulrahman Jairoun et al. [36]. Furthermore, the reports by Dehua Hu [37] et al. and Jakob Weitzer et al. [26] showed that a few friends and family members of participants (12%) also expressed substantial VBH. There was the lowest incidence of VBH when family members were already vaccinated or were willing to take the vaccine.

Pregnant women (30%) expressed significantly higher VBH than non-pregnant women (6.8%) as per Sameh Attia et al.’s [34] study. Similarly, Elias Kowalski et al. [31] found that participants (both gender) without children showed greater hesitancy than those with children. Tesfaye Yadete et al. also observed that populations expressing higher VBH did not intend to have children of their own [17].

Kavita Batra et al.’s [19] study found significantly greater VBH among single and unmarried participants than among married participants (33.0% vs. 24.3%, *p* = 0.04). Tesfaye Yadete et al. [17] also reported similar observations.

There are conflicting reports regarding the effect of religion on VBH. Jakob Weitzer et al. [26] reported that those who rarely participated in religious activities expressed lower VBH. On the contrary, religiously unaffiliated participants expressed greater VBH, according to Tesfaye Yadete et al. [17].

Ryan C. Lee et al. [38] report that black respondents scored much lower on the mean trust in science scale than all other races. This is supported by Neil G Bennett et al. [20] who found lower VBH among Asians and Hispanics. Quite by contrast, Kimberly H. Nguyen et al. [15] reported the highest VBH among Non-Hispanic black and Hispanic adults.

Improved quality of lifestyle has caused people to gain weight. There was a single study that linked obesity and VBH. Obese adults expressed less VBH, according to Mohamed Abouzid et al. [18].

The employment status of the participant had contributed to VBH. Ammar Abdulrahman Jairoun et al. [36] found that the unemployed expressed less VBH than employees in both non-health and health sectors. This is supported by Kimberly H. Nguyen et al. [15], Shyam Raman et al. [39], and Jakob Weitzer et al. [26]. However, Gede Benny Setia Wirawan et al. [21] found that policy changes in work environment, diminished VBH. In a study by Sameh Attia, students expressed greater VBH than those employed [33].

Lower socio-economic background of the participants was associated with uncertainty and VBH, according to Elise Paul et al. [22]. The majority of those expressing VBH belonged to rural areas, as per Mohamed Abouzid et al.’s [18]. Additionally, Kimberly H. Nguyen et al. [15] reported that booster vaccinations were lowest among low-income adults. However, a Malaysian study by Thin Mon Kyaw et al. [32] reported greater VBH with higher income (≥5000 MYR).

##### Psychological Factors

Most respondents expressing VBH believed that vaccines would evoke negative side effects or harm as per Mohamed Lounis et al. [24], Marine Paridans et al. [40], Neil G Bennett et al. [20], Makoto Yoshida et al. (57.7% concerned about adverse effects) [41], Dehua Hu et al. [37], and Gede Benny Setia Wirawan et al. [21]. Similarly, Petros Galanis et al. [16] found that possible short (29%) and long-term side effects (46.8%) were the main reasons for VBH or a new COVID-19 vaccine. On the other hand, Thin Mon Kyaw et al. [30] found that respondents were neutrally (36.0%) worried about serious adverse reactions after vaccination.

VBH was inversely correlated to trust in science as shown by Massamiliano Baratucci et al. [42]. People who believed in conspiracy theories expressed greater VBH, according to Elias Kowalski et al. [30] and Neil G Bennett et al. [20]. Asians exhibited greater confidence in science than whites, which correlated with higher odds (OR = 8.73) of accepting the vaccine booster. Black respondents scored much lower on the mean trust in science scale than all other racial/ethnic groups as reported by Ryan C. Lee et al. [38].

34.7% believed that vaccinations were unnecessary because COVID-19 outbreak in China was under control, as illustrated by Chenyuan Qin et al. [14]. Mohamed Abouzid et al. [18] reported that participants believed booster doses were superfluous (14.3%, n = 436). Likewise, Kevin Y. K. Tan et al. [35], Stephen R Neely [43] (29%), and Petros Galanis et al. [16] (19.4%) supported the above findings. Some believed they took the previous dose only a while ago and the next dose was unnecessary (24.6%). As per Walid Al Qareem et al. [44], this was the most cited reason for VBH [44]. Another common reason for VBH was the unavailability of recommendations (10.5%) for the booster dose after the initial COVID-19 shot, as Lai et al. [45] reported.

Chenyuan Qin et al. [11] showed that 30.2% believed that one to two doses were enough, supported by Mohamed Lounis et al. [24]. Likewise, 25% in Makoto Yoshida et al.’s [41] study believed that additional doses were unnecessary. Sameh Attia et al. [34] reported that those who received one dose of COVID-19 vaccine expressed substantially higher VBH (62.2%) than those who received two or three doses (91.2% and 98.3%, respectively; *p* < 0.001) [33]. Martin S Hagger et al. [46] and Kyra’s model for predicting VBH demonstrated that the social cognition construct has a major role in VBH, which also depends on people’s attitude and subject norms.

In a study by Chenyuan et al. [14], among 196 participants who expressed VBH, 58.4% and 50.0% were unsure of vaccine’s effectiveness and safety. This is also supported by Mohamed Abouzid et al. [18] (14.6% hesitant), Thin Mon Kyaw et al. [30], Piotr Rzymyski et al. [32] (22.4% hesitant), Xiaozhen Lai et al. [45], Abdul Moeed et al. [29], Marine Paridans et al. [40], Petros Galanis et al. [16], Lucio folcarelli et al. [47] (43.2% hesitant), Makoto Yoshida et al. [41] (19.2% hesitant), and Xiaozhen Lai et al. (10.2% hesitant due to efficacy concerns and 11.8% hesitant due to safety concerns) [45]. Stephen R Neely [43] showed that one in ten did not think immunization stopped the spread of COVID-19 (10.0%).

The marginal mean willingness to get a booster dose for preventing 50% symptomatic infection was only 0.49. A booster with 70% of the effect increased the marginal mean willingness to 0.59. For 90% success, the marginal mean willingness further increased to 0.73. Marginal means for all three degrees of efficacy differ from one another significantly, portrayed by Shyam Raman et al. [39].

##### Infection

Lower stress levels generated by concerns of infection or becoming seriously ill also boosted VBH. A study in Pakistan by Abdul Moeed et al. [29] showed that VBH was related to COVID-19 being considered as a seasonal flu (26.4%) and belief in natural immunity (22.9%). In Qin et al.’s [14] study, 14.4% believed they were healthy enough to fight COVID-19. This was also supported by Elise Paul et al. [22]. Yufang Sun et al. [48] reported that the main reason for VBH was the low perceived risk of infection with COVID-19. A US study by Stephen R Neely et al. reported that fewer than one in ten respondents were never concerned about COVID-19 (12.3%) [43]. Similarly, in a cross-sectional study by Petros Galanis et al. [16], 19.4% felt that they did not require any vaccine because they enjoyed adequate immunity to COVID-19.

Mohamed Abouzid et al. [18] found the lowest VBH among those who weren’t infected with COVID-19. Quite in contrast, Sameh Attia et al. [34] suggested that those with a history of infection (76.4%) expressed significantly (*p* < 0.01) greater VBH. Similarly, Carlos Izaias Sartorão-Filho et al. [49] showed that people infected with COVID-19 were 5.4 times more likely to express VBH. Moreover, Yufang Sun et al. [48] and Gede Benny Setia Wirawan et al. [21] suggested that rapid viral mutation and strain changes were reasons for VBH.

##### Vaccine

One of the major reasons for VBH was side effects associated with previous COVID-19 vaccine doses (10.3% of respondents), according to Mohamed Abouzid et al. [18]. Carlos Izaias Sartorão-Filho et al. [49] stated 4.7 times more VBH due to a history of side effects. Jairoun et al. [36]’s study showed 35.1% of participants expressing concern due to negative side effects, whereas 65.5% were worried about unforeseen side effects and 47.3% were hesitant due to general mistrust. The history of side effects being a reason for VBH was also supported by Stephen R Neely [43] and Sameh Attia et al. [33].

Studies by Tesfaye Yadete et al. [17], Ammar Abdulrahman Jairoun et al. [36], Xiaozhen Lai et al. [45], Mohamed Abouzid et al. [18] suggested that people who did not receive the first dose of COVID-19 vaccine, and were not used to vaccination against influenza, expressed greater VBH. Similarly, Sky Wei Chee Koh et al. [50] showed that those who were hesitant during their first dose expressed 3.66 times greater VBH.

A study by Khalid Alhasan et al. [51] in Saudi Arabia reported a lower preference for the AstraZeneca vaccine, whereas Mohamed Lounis et al. [24] found the most preferred COVID-19 vaccine to be Sinovac (33.3%), followed by Janssen (12.6%), AstraZeneca-Oxford (11.8%), and Pfizer-BioNTech (9.6%). However, in the US, Shyam Raman et al. [39] showed that marginal mean willingness was highest for Pfizer, followed by Moderna, and, finally, Johnson & Johnson. VBH due to preference of vaccine was also shown by Sameh Attia et al. [34] where 15.8% of participants wished to receive a different type of vaccine, of which some wanted the government to purchase a certain type such as BNT 16B2. mRNA-1273, AZD1222, and Ad26.COV2. S.

##### Social Media

Another factor identified was media consumption.

Gede Benny Setia Wirawan et al. [21] found that VBH were negatively correlated with trust in reliable information sources. However, print media’s influence was favorably linked to acceptance, while television’s influence was found to boost VBH.

##### Healthcare/Non-Healthcare Staff

Fan Wu et al.’s [31] study showed that males and medical personnel expressed less VBH than females and non-medical employees. Sky Wei Chee Koh et al. [50] found that administrative healthcare workers expressed greater VBH than ancillary, medical, and nursing staff.

However, Mohamed Lounis et al. [24] demonstrated that healthcare professionals expressed greater VBH (*p* = 0.011) (45.9%) than non-medical professionals, which has been supported by Xiaoxiao Wang et al. [23].

##### Governmental Policy

Shyam Raman et al. [39], Kavita Batra et al. [19], Tesfaye Yadete et al. [17], and Neil G Bennett et al. [20] showed that Republicans expressed greater VBH than Democrats. Ruben Juarez et al. [52]’s study underlines lack of faith in official information and sources significantly aggravated VBH. However, Marine Paridans et al. [40] stated that hesitant groups in their study were not aligned with the vaccination plan.

Other factors associated with VBH were a lack of trust in the government, low compliance with COVID-19 government guidelines, and right-leaning political views.

## 4. Quality Assessment

The quality of the articles included in the review was thoroughly assessed. The quality of the results of the systematic review directly depends on the overall strength of the evidence collected. By utilizing the Critical Appraisal Skills Program (CASP) checklist, all articles selected for the review were of good quality, with definitive research idea, appropriate aim, methodology and findings. The only difficulty was to check for an association between the researcher and participant of the study followed by determining whether the ethical considerations were taken into account. Eight of the 10 questions of the CASP yielded a definitive ‘yes’ for each article, thereby leading us to conclude that all articles included in the systematic review were of high quality and are highly reliable (Supplementary Data S2).

## 5. Discussion

This comprehensive systematic review provides an overview of the factors contributing to VBH. The review included a total of 42 studies representing 28 countries, with a predominant representation from the USA (N = 9), followed by China (N = 9), and three studies each from Germany and India. Most of the remaining countries were represented by only one study. Although the included studies represent countries from almost all continents, sub-Saharan Africa is underrepresented. From another perspective, around 17 studies were conducted in Asian countries while 10 were European studies. The remaining were conducted in North America (N = 10), the Middle East and North Africa (N = 6), and Latin America (N = 1).

Factors evaluated may be grouped under three main categories and a range of individual attributes. The three main categories are sociodemographic, financial, and psychological factors. Individual attributes are related mainly to the impact of social media, government policy, and perceptions toward vaccine efficacy and safety. Most studies have reported factors belonging to at least two groups of VBH determinants, indicating the need for customized interventions to target the modifiable factors contributing in every context.

There are very few studies conducted in India confined to VBH. However, considering the population of India and its unique demographics, multiple reasons for VBH were revealed in comparison to the vast majority of reasons cited commonly by people across the world. In one of the studies, the most frequently mentioned reasons for VBH were lack of vaccination slots, vaccine doses or centers being too far. These reasons for VBH were uniquely limited to India. Similarly, believing COVID-19 doesn’t exist anymore, belonging to rural areas, or not knowing others tested positive for COVID-19 were other reasons cited primarily in India, which reflects the outstanding cultural diversity and demographic mass that characterizes the Indian population.

Meanwhile, among Japanese populations, VBH was positively correlated with younger age and influenced by perceptions of vaccine safety and efficacy [50]. The application of protection motivation theory among the Chinese population shows that VBH was associated with high perceived severity and response cost [31]. A Singapore study revealed that VBH was lower than first-dose hesitancy among healthcare workers. Those expressing first-dose hesitancy were more likely to express VBH [50]. Additionally, another study in Singapore reported VBH in about a third of the study population expressing lower threat perceptions, lower perceived benefits, and higher perceived concerns [32]. Therefore, it is essential to consider focused interventions to restrain VBH and facilitate vaccine-related health perceptions.

Furthermore, a large Chinese study (n = 6375) revealed a comprehensive list of demographic and psychosocial factors that mitigate VBH. Lower VBH is reported among younger age groups, females, recipients of higher education, those suffering fewer side effects with previous doses, those with higher perceived susceptibility, infection severity, and finally, a greater trust level in the authorities [53]. Thus VBH is multifactorial, with potentially modifiable factors with appropriate interventions. In addition to socio-psychological factors that lower VBH, trust in science can significantly elicit vaccination intention [42]. In a Malaysian study (n = 1010), lower VBH was associated with younger age, higher income, Chinese ethnicity, and fewer previous experiences of side effects [54]. Knowledge about and confidence in COVID-19 vaccines and trust in the government diminishes VBH among middle-eastern migrants in Australia [55]. Reports from culturally diverse communities suggest the need for deploying tailored interventions that address separate, context-specific VBH determinants.

US data indicate that VBH is higher among those who distrust vaccine efficacy or the government. On the other hand, VBH was lower among those who regularly took their seasonal flu vaccine or feared potential job losses [20]. Moreover, another study among healthcare workers in the US underlined that their primary motivation for booster doses was impelled by their desire to protect themselves and others. Meanwhile, the study highlighted vaccine-related safety concerns and misinformation, amplified by race-related medical mistrust and a lack of proper communication, which become significant determinants of VBH [56]. Therefore, interventions or campaigns that neutralize VBH should focus on major safety concerns and overcome barriers to trust and communication between different races. A Latin American study underpinned that VBH is positively correlated with low education, living in a town, food insecurity, depressive symptoms, and a previous COVID-19 infection. VBH was negatively correlated with being female and having anxiety symptoms [57].

A Polish study found that VBH was higher among those who experienced side effects after previous doses, expressed safety concerns, and did not perceive the need for further vaccination. Lower VBH was reported among older adults, obese women, people with chronic diseases, and those who had previous influenza vaccine experiences [32]. Moreover, a study from Italy showed that VBH was more likely among those with unpleasant experiences after previous vaccination, lack of close family or friends infected by COVID-19, and a dearth of official information from government authorities. Furthermore, a large study (n = 22,139) in the UK has highlighted that approximately 4% were uncertain and another 4% expressed VBH. The uncertainty or unwillingness was greater among those who had the same attitude towards the first dose: younger, healthier, low education, disadvantaged socio-economics, low perceived susceptibility, and low compliance to government-imposed COVID-19 restrictions [47].

Many reports have confirmed the multifactorial nature of vaccine hesitancy, necessitating multifaceted, tailored interventions that address all potentially modifiable factors [49]. Indeed, customized education, proper communication on perceived risks and benefits, and strategic legislation might help reduce VBH among HC workers [50]. Interestingly, a series of ten cross-sectional studies in Hong Kong (n = 7411) revealed that factors associated with VBH change over time, mandating the need to update and adjust vaccine promotion strategies in the community [58]. Any intervention to increase booster vaccine uptake should not overlook previous experiences following the first two doses. Unaddressed adverse experiences after primary doses can consistently impact VBH even among healthcare workers [59]. Previous research shows that vaccine-related side effects vary across age groups, gender, and recipients of different vaccines [60]. These differences add another dimension when considering interventions to counter VBH, where the above groups should be carefully assessed and counseled accordingly.

Evaluating these articles demonstrated that the major factors for VBH were in the domain of trust deficit in the vaccine, its efficacy, science, or policy. In a vastly populated country like India, despite multiple government schemes for vaccination, such as HarGharDastak or 75 days of free vaccination (in connection with 75th year of Independence), it is necessary for these schemes to reach the public. We need to understand the level of awareness of these schemes among the common multitude. This review aims to facilitate the spread of awareness and bridge the communication gap between science and the people.

## 6. Conclusions

In our study, we found strong evidence suggesting age, gender, lack of trust in science, and concerns of safety and efficacy as major determinants for VBH. Among other factors, employment status, infection with COVID-19, fear of side effects, and governmental policies were identified. Identifying these factors can help shape interventions that improve the vaccination strategy. Targeted intermediation based on specific reasons for VBH can facilitate purposeful interventions. As the main message of this systematic review, we propose a thorough evaluation of factors that potentially affect hesitancy, followed by appropriate communication at both individual and community levels about the benefits of booster doses and the risk of losing immunity through their neglect.

## Data Availability

Not applicable.

## Supplementary Materials

The following supporting information can be downloaded at: https://www.mdpi.com/article/10.3390/vaccines11030623/s1, Suplimentary Data S1: Data extracted from the included articles; Suppleimentary Data S2: The quality of each included article was assessed through the Critical Appraisal Skills Program (CASP) checklist.
