# Supplementary material for: A Systematic Review on Sociodemographic, Financial and Psychological Factors Associated with COVID-19 Vaccine Booster Hesitancy among Adult Population
Source: Vaccines (Basel). 2023 Mar 9;11(3):623. doi: 10.3390/vaccines11030623 (PMC10051942; doi:10.3390/vaccines11030623)
Supplement: Supplementary file 1 [file vaccines-11-00623-s001.zip › vaccines-2222518-Supplementary.pdf]

Suplimentary Data S1: Data extracted from the included articles.

Sl. no	Author, year	Country of origin	Age (years) of participants	Sample size	Study design	Enrollment method	Contributing Factors for COVID-19 vaccine hesitancy
1.	Chenyuan Qin et al [14] 2022	China	>18 years	3119	Cross sectional study	Random allocation of the questionairre among 31 selected provinces on the platform Wen Juan Xin.	• Age<br>• Safety and efficacy<br>• Belief in health, COVID-19 vaccination process and infection, number of doses
2.	Fan Wu et al. [31] 2022	China	>18 years <80 years	8229	Cross sectional study	Snowball sampling	• Gender<br>• Profession<br>• Time gap after first dose.<br>• Age<br>• Education<br>• News
3	Dehua Hu et al 2022 [37]	China	>18 years	898	Web based, online cross-sectional study,	Snowball sampling	• Type of vaccine and manufacturer<br>• Adverse effects<br>• Readiness of friends and family
4	Xiaoxiao Wang et al, 2022 [23]	China	>18 years	2047	Cross sectional study	Snowball sampling	• Age<br>• Gender<br>• Profession<br>• Education
5	Yufang Sun et al, 2022 [48]	China	>18 years	1,062	Cross-sectional online survey	Snowball sampling	• Low perception of infection danger<br>• SARS-CoV-2 fast mutation
6	Ruitong Wang, 2022 [28]	China	>18 years	3,242	web-based cross-sectional survey,	Participants randomly selected in Wen Juan Xin database accoring to inclusion crtiera.	• Social media<br>• Traditional media
7	Xiaozhen Lai et al, 2022 [45]	China	>18 years	1167	Cross-sectional study/	Participants randomly selected in Wen Juan Xin database accoring to inclusion crtiera.	• Safety concerns<br>• Unaware about the booster dose/ not recommended by their physician .<br>• No need for booster<br>• Efficacy concern
8	Tesfaye Yadete et al, 2021 [17]	USA	>18 years	2138	Cross sectional study,	Participants randomly selected by Qualtrics according to the inclusion criteria.	• Age<br>• Previously unvaccinated<br>• Fertitlity concern.<br>• Gender.<br>• Political affiliation<br>• Religious affiliation<br>• Insurance<br>• Education<br>• Family with vulnerable health conditions/ prior COVID-19 infection.
9	Shyam Raman et al, 2022 [39]	USA	>18 years	2241	Cross sectional study,	Quota based sampling using Lucid.	• Age<br>• Employment<br>• Previously unvaccinated<br>• Political affiliation<br>• Vaccine brand<br>• Efficacy
10	Kavita Batra et al, 2022 [19]	USA	>18 years	501	Cross sectional study,	Participants randomly selected by Qualtrics according to the inclusion criteria.	• Previously unvaccinated<br>• Age<br>• Marital status<br>• Political affiliation<br>• Education
11	Ryan C. Lee et al 2022 [38]	USA	>18 years	5256	Cross sectional study,	Participants who enrolled in TPRI health cohort.	• Never infected with COVID-19<br>• Trust deficit in science.<br>• Race
12	Neil G. Bennett et al, 2022 [20]	United states	>18 years	3497	Cross sectional, survey-based study,	Quota based sampling using Qualtrics.	• Age<br>• Lack of trust<br>• Political affiliation<br>• Flu vaccine habits<br>• Perception of being at high risk<br>• Race
13	Kimberly H. Nguyen et al, 2022 [15]	USA	18-65 years	74,995	Cross-sectional study/	Not specified	• Race<br>• Education<br>• Income<br>• Insurance<br>• History of COVID-19<br>• Employment
14	Massamiliano Baratucci et al, 2022 [42]	USA	-	1095	Cross sectional study	Participants randomly selected by Qualtrics according to the inclusion criteria.	• Gender.<br>• Employment<br>• Socioeconomic status<br>• Religious affiliation
15	Stephen R Neely ,2022 [43]	USA	>18 years	600 adults	Cross sectional study,	Quota sampling	• Booster is not needed<br>• Side effects concern<br>• Not afraid of 19 infection

							• Belief that booster shots would be an inconvenience<br>• No belief in vaccine
16	Martin S Hagger et al, 2022 [46]	USA	>18 years	479	Cross sectional study	Participants randomly selected by Qualtrics according to the inclusion criteria.	• Political ideology<br>• Free will belief<br>• Efficacy<br>• Risk perception
17	Mostafa Saidur Rahim Khan et al, 2022 [25]	Japan	>20 years	2912	Cross sectional study	Used panel data from the Household Behavioral and Financial Survey, Japan.	• Concerns of future effect on health status<br>• Marital status
18	Makoto Yoshida et al 2022 [41]	Japan	>18 years<br><br>12-19 years	2439<br><br>58	Retrospective cohort,	Paper based questionnaire recruiting a homogenous japanese rural population from Fukushima Prefecture, including Ishikawa gun, Soma City, and Minamisoma City.	• Two doses are sufficient<br>• Fear of ADRs<br>• Unknown safety and efficacy.<br>• Age
19	Sky Wei Chee Koh et al 2022 [50]	Singapore	>18 years	891	Retrospective observational study	Healthcare workers from singapore public primary heatlhcare workers.	• Hesitancy during first dose<br>• Healthcare/non healthcare
20	Kevin Y. K. Tan et al 2022 [35]	Singapore	>18 years	1552	Cross sectional, survey-based study,	Door to door recruitment by randomly selecting an equal number of of households in 5 geographical zones in singapore.	• Negative belief on efficacy<br>• Education<br>• Poor understanding of the need for booster vaccinations<br>• Lower perception on COVID-19 risks
21	Elias Kowalski et al. 2022 [30]	Germany	>18 years	224	Cross sectional study	All patients with a positive SARS-CoV-2 PCR result reported to the Freudenstadt Health Department	• Gender<br>• With/without children<br>• Belief in government<br>Belief in conspiracy theories
22	Sameh Attia et al, 2022 [34]	Germany	>18 years	930	Cross sectional study/	Self administered questionnaire circulated digitally using KoBo toolbox	• Gender<br>• Pregnancy status<br>• Previously infected<br>• Previously unvaccinated<br>• Previously received not more than one dose of vaccine<br>• Experienced serious post-vaccination or moderate side effects<br>• Employment<br>• Type of vaccine
23	Jakob Weitzer et al, 2022 [26]	Germany, Austria, Switzerland	>18 years	2,480	Cross sectional study,	participants were recruited by "open enrollment" and "by invitation only" campaigns via email and online marketing channels	• Age<br>• Education<br>• Residing in rural areas
24	Geetanjali C. Achrekar et al. 2022 [11]	India	>18 years	687	Cross sectional online survey,	The survey was distributed via the Qualtrics platform, and the survey link was distributed to a wide range of communities, research investigators' social networks, social media, and other networking platforms, including WhatsApp, Facebook, etc.	• Previously unvaccinated<br>• Income<br>• Residing in rural or urban area<br>• Belief that booster dose had more side effects<br>• Vulnerable population or family previously COVID-19 infected
25	Masthi NR R et al. 2022[12]	India	>18 years	550	Cross sectional online survey	Convenient sampling was used to collect responses. Snowball sampling technique was used to recruit participants.	• Not yet due for booster dose<br>• Non-availability of vaccine<br>• Side effects after the initial doses
26	Sajith Vellappally et al, 2022 [13]	India and Saudi Arabia	>21 years	530-India and 303-SA	Cross sectional study	Convenient sampling method was used to collect responses. Healthcare workers from both countries were asked to participate.	**India :**<br>• Efficacy<br>• Concerns about long term adverse effects<br>**Saudi arabia:**<br>• Poor understanding about immunization<br>• Concerns about long term adverse effects
27	Khalid Alhasan et al 2021 [51]	Saudi Arabia	>18 years	1279	Cross sectional study	Healthcare workers invited through convenient sampling technique.	• Type and manufacturer
28	Mohamed Abouzid et al. 2022 [18]	Middle east and north Africa	>18 years	4056	Cross sectional study	Not specified. Survey was distributed to varrious communities through various channels.	• Safety uncertainties<br>• Belief that vaccine booster was unnecessary<br>• Type of previous vaccine taken.<br>• Gender<br>• Obesity<br>• Individuals who often receive influenza vaccine<br>• Previously uninfected

							• Previous side effects
29	Ammar Abdulrahman Jairoun et al. 2022 [36]	UAE	>18 years	614	Cross sectional, online survey,	Random selection using self administered questionnaire.	• Employment status<br>• Family previously COVID-19 infected<br>• Previously unvaccinated
30	Walid Al Qareem et al. 2022 [44]	Jordan	18 years and above.	1144	Cross sectional study,	Questionnaires made on google forms were circulated on Jordanian all purpose facebook groups.	• Trust deficit in science and efficacy<br>• Belief that there is no need for booster dose<br>• Belief that already infected with 19 so there isn't a need for a shot
31	Mohamed Lounis et al. 2022[24]	Algeria	>18 years	787	Cross sectional, survey-based study	self-administered questionnaire (SAQ) created and distributed online using Google Forms.	• Belief that two doses are sufficient<br>• Inefficiency of vaccine<br>• Fear of adverse effects and health hazard to immune system.<br>• Healthcare/non healthcare workers<br>• Manufacturer and type of vaccine.
32	Abdul Moeed et al, 2022 [29]	Pakistan	- 77% were 18-30 years	787	Cross-sectional web-based survey.	Data collected b y circulation of google forms on social media platforms.	• Age<br>• Safety worries brought on by side effects from prior doses<br>• The seasonal flu-like nature of COVID-19<br>• Belief in immunity from nature<br>• Brand of vaccine
33	Thin Mon Kyaw et al 2022[32]	Malaysia	>18 years	467	Cross sectional study,	Convenient sampling technique by circulation of google forms on social media platforms.	• Gender<br>• Booster dose efficacy against newer strains of COVID-19.<br>• Income
34	Gede Benny Setia Wirawan et al 2022[21]	Indonesia	>18 years	3088	Cross sectional survey-based study,	Online survey with geolocation filtering.	• Perception of obstacles and harm<br>• Lack of trust in information source<br>• Social media<br>• Print media<br>• Telivision influence.<br>• New strains of COVID-19.
35	Piotr Ryzmyski et al 2021. [33]	Poland	>18 years	2782	Cross sectional study,	Snowball sampling using a structured self designed online questionnaire.	• Gender<br>• Side effects scare<br>• People who got SARS COV 2 after one dose of vaccine.
36	Miloslav Klugar et al 2021[27]	Czechia	>18 years	3454	Cross sectional, survey-based study	Self adminitered questionnaire disseminated through KoBo toolbox	• Gender.<br>• Age.<br>• Healthcare/non healthcare
37	Ruben Juarez et al 2022 [52]	USA	>18 years	1594	Cross sectional study	Online survey using Qualtrics	• Trust and consumption of official sources of information.
38	Marine Paridans et al, **2022** [40]	Belgium	>18 years	1030	Cross sectional study	Self administered questionnnaires distributed using online platforms.	• Disapproval of the vaccination plan<br>• Efficacy<br>• Concerns regarding harmfulness
39	Petros Galanis et al 2022 [16]	Greece	Mean age of 37 years.	815	Cross-sectional study	Convenient sampling by creating an anonymous questionnaire on google forms and disseminating them through social media platforms and personal emails.	• Worried about safety<br>• Effectiveness<br>• Low perceived risk of consequences with COVID-19.<br>• Considering no need for booster.<br>• Tired of the vaccination process
40	Elise Paul et al 2022 [22]	UK	>18 years	22,139 (fully vaccinated adults)	Cross-sectional study	Stratified into 3 approaches:<br>a. Convenience sampling<br>b. Targeted recruitment<br>Promoted by partnership organizations to the vulnerable populations	• Age<br>• Low education<br>• Low socio-economic position<br>• Healthy adults<br>• No fear of COVID 19<br>• Low levels of present stress related to contracting or becoming dangerously ill from COVID-19
41	Lucio folcarelli et al 2022 [47]	Italy	>18 years	615	Cross sectional study	Systematic random sampling from the regustered second dose subjects.	• Concerns about safety (43.2%)<br>• No need for booster after 2 doses (27.3%)
42	Carlos Izaias Sartorão-Filho et al, 2022 [49]	Brazil	>18 years	347	Cross sectional study	Personal text messages and emails to all participants due for second dose for over a month.	• Age<br>• History of COVID-19 infection<br>• History of adverse effects

TPRI: Trojan Pandemic Response Initiative

***Suppleimentary Data S2*:** The quality of each included article assessed through the Critical Appraisal Skills Program (CASP) checklist.

Articles	CASP Questions									
	**1**	**2**	**3**	**4**	**5**	**6**	**7**	**8**	**9**	**10**
Chenyuan Qin et al, 2022	Y	Y	Y	Y	Y	C	Y	Y	Y	Y
Fan Wu et al, 2022	Y	Y	Y	Y	Y	C	C	Y	Y	Y
Elias Kowalski et al, 2022	Y	Y	Y	Y	Y	C	C	Y	Y	Y
Tesfaye Yadete et al, 2021	Y	Y	Y	Y	Y	C	C	Y	Y	Y
Geetanjali C. Achrekar et al, 2022	Y	Y	Y	Y	Y	C	Y	Y	Y	Y
Ammar Abdulrahman Jairoun et al, 2022	Y	Y	Y	Y	Y	C	Y	Y	Y	Y
Mohamed Abouzid et al, 2022	Y	Y	Y	Y	Y	C	C	Y	Y	Y
Masthi NR R et al, 2022	Y	Y	Y	Y	Y	C	C	Y	Y	Y
Shyam Raman et al, 2022	Y	Y	Y	Y	Y	C	C	Y	Y	Y
Kavita Batra et al, 2022	Y	Y	Y	Y	Y	C	Y	Y	Y	Y
Ryan C. Lee et al, 2022	Y	Y	Y	Y	Y	C	C	Y	Y	Y
Khalid Alhasan et al, 2021	Y	Y	Y	Y	Y	C	C	Y	Y	Y
Mohamed Lounis et al, 2022	Y	Y	Y	Y	Y	C	Y	Y	Y	Y
Miloslav Klugar et al, 2021	Y	Y	Y	Y	Y	C	Y	Y	Y	Y
Sky Wei Chee Koh et al, 2022	Y	Y	Y	Y	Y	C	Y	Y	Y	Y
Kevin Y. K. Tan et al, 2022	Y	Y	Y	Y	Y	C	C	Y	Y	Y
Ruben Juarez et al, 2022	Y	Y	Y	Y	Y	C	C	Y	Y	Y
Dehua Hu et al, 2022	Y	Y	Y	Y	Y	C	C	Y	Y	Y
Makoto Yoshida et al, 2022	Y	Y	Y	Y	Y	C	C	Y	Y	Y
Thin Mon Kyaw et al, 2022	Y	Y	Y	Y	Y	C	Y	Y	Y	Y
Neil G. Bennett et al, 2022	Y	Y	Y	Y	Y	C	C	Y	Y	Y
Gede Benny Setia Wirawan et al, 2022	Y	Y	Y	Y	Y	C	Y	Y	Y	Y
Piotr Resmyski et al, 2021	Y	Y	Y	Y	Y	C	C	Y	Y	Y
Jakob Weitzer et al, 2022	Y	Y	Y	Y	Y	C	C	Y	Y	Y
Walid Al Qareem et al, 2022	Y	Y	Y	Y	Y	C	C	Y	Y	Y
Xiaoxiao Wang et al, 2022	Y	Y	Y	Y	Y	C	C	Y	Y	Y
Abdul Moeed et al, 2022	Y	Y	Y	Y	Y	C	C	Y	Y	Y
Yufang Sun et al, 2022	Y	Y	Y	Y	Y	C	C	Y	Y	Y
Kimberly H. Nguyen et al, 2022	y	Y	Y	Y	Y	C	C	Y	Y	Y
Massamiliano Baratucci et al, 2022	Y	Y	Y	Y	Y	C	C	Y	Y	Y
Marine Paridans et al, 2022	Y	Y	Y	Y	Y	C	C	Y	Y	Y
Ruitong Wang, 2022	Y	Y	Y	Y	Y	C	C	Y	Y	Y
Stephen R Neely ,2022	Y	Y	Y	Y	Y	C	C	Y	Y	Y
Xiaozhen Lai et al, 2022	Y	Y	Y	Y	Y	C	C	Y	Y	Y
Sameh Attia et al, 2022	Y	Y	Y	Y	Y	C	C	Y	Y	Y
Petros Galanis et al, 2022	Y	Y	Y	Y	Y	C	C	Y	Y	Y
Elise Paul et al., 2022	Y	Y	Y	Y	Y	C	C	Y	Y	Y
Lucio folcarelli et al, 2022	Y	Y	Y	Y	Y	C	C	Y	Y	Y
Carlos Izaias Sartorão-Filho et al, 2022	Y	Y	Y	Y	Y	C	C	Y	Y	Y
Martin S Hagger et al, 2022	Y	Y	Y	Y	Y	C	C	Y	Y	Y
Walid Al-Qerem, et al, 2022	Y	Y	Y	Y	Y	C	C	Y	Y	Y
Mostafa Saidur Rahim Khan et al, 2022	Y	Y	Y	Y	Y	C	C	Y	Y	Y
Sajith Vellappally et al, 2022	Y	Y	Y	Y	Y	C	C	Y	Y	Y

1. Was there a clear statement of the aim of the research?;
2. Is a qualitative methodology appropriate?;
3. Was the research design appropriate to address the aims of the research?
4. Was the recruitment strategy appropriate to the aims of the research?
5. Was the data collected in a way that addressed the research issue?
6. Has the relationship between the researcher and participants been adequately considered?
7. Have ethical issues been taken into consideration?
8. Was the data analysis sufficiently rigorous?
9. Is there a clear statement of findings?
10. How valuable is the research?

Abbreviation: Y-Yes, N-No, C- Cannot tell.
